# Non-pharmacological interventions of pain management used during labour; an exploratory descriptive qualitative study of puerperal women in Adidome Government Hospital of the Volta Region, Ghana

**DOI:** 10.1186/s12978-021-01141-8

**Published:** 2021-04-23

**Authors:** Kennedy Diema Konlan, Agani Afaya, Eugenia Mensah, Amos Nawunimali Suuk, Dahamata Issahaku Kombat

**Affiliations:** 1 Department of Public Health Nursing, School of Nursing and Midwifery, University of Health and Allied Sciences, Ho, Ghana; 2 Department of Nursing, School of Nursing and Midwifery, University of Health and Allied Sciences, Ho, Ghana; 3 College of Nursing, Yonsei University, 50-1, Yonsei-ro, Seodaemun-gu, Seoul, 03722 Korea; 4 War Memorial Hospital, Navrongo, Upper East Region Ghana; 5 Community Health Nursing Training College, Tamale, Northern Region Ghana; 6 College of Nursing and Midwifery, North East Region, Nalerigu, Ghana

**Keywords:** Pain, Childbirth, Management, Nonpharmacological, Labour, Puerperal

## Abstract

**Background:**

Women have experienced labour pain over the years as various attempts have been made to effectively manage this pain. There is paucity of literature on the experiences and perceptions about labour pain management with the contemporary Ghanaian health system. This study explored the perspective of puerperal women on the use of non-pharmacological labour pain management at Adidome Government Hospital.

**Methods:**

The study adopted an exploratory descriptive qualitative approach as data was collected through individual interviews. Informed consent was obtained from all participants who were purposely sampled until data saturation was reached on the 17th participant. Interviews were audio recorded and transcribed immediately. Thematic analysis was engaged in three interrelated stages, namely data reduction, data display, and data conclusion to analyse the transcript and field notes. Results were presented with supporting quotes from the transcripts.

**Results:**

The women described labour pain as very severe, severe and moderate as the pain lasted more than 12 h. The various strategies adopted in managing labour pains included shouting and walking around, crying and screaming and staying calm and snapping the fingers. Other pain management strategies adopted during labour included women engaged in deep breathing exercises, chatting with other people and relatives, diversion therapy, reassurance, taking a shower, assuming side lying positions, and receiving intravenous therapy. The presence of the husband of a labouring woman during labour improved pain bearing ability.

**Conclusion:**

It is important that midwives institute pragmatic protocols in the labour ward that ensure a relaxing atmosphere for women in labour, respond to the sensitivity and specificity of labouring women needs and when possible significant others (e.g., husband) of the labouring women could be allowed to visit. Labour wards should be made sound proof to allow women the ability to express themselves satisfactorily during labour without fear of being heard outside.

## Plain Language Summary

During childbirth, women have complained of severe pains as health personnel over the years keep looking for appropriate means to alleviate the pain. We therefore studied women with children less than 42 days to understand their perspective on the use of non-drug methods in relieving pain during birth. With the intention of exploring this phenomenon we had an interview with 17 women in the Adidome government hospital. The women showed that labour is a very painful experience. The methods they adopted in reducing pain included shouting, walking around the hospital, crying or screaming, staying calm and snapping the fingers. These women were generally happy with the care that was rendered by midwives. Other methods used to reduce pain were taking deep breaths, talking with a family member or friend, taking a bath, assuming a lying position and receiving intravenous infusion. Women who had their husband present during labour said it helped their pain endurance. We therefore suggested that midwives should be encouraging and supportive of women who are in labour and if possible, allow their spouses into the birth room to augment the pain adaptation ability. Also, labour wards should have sound resilient so that women can make any sound if they so wish to.

## Introduction

Labour is an inevitable physiological process that most women do not want to think of because of the bad experience they face largely due to the pains that accompany it [1]. Labour is mostly considered to be one of the most painful experiences in the life of women [1, 2]. Pain is described as any physical discomfort caused by illness or any physical injury that can greatly impact one’s daily life activities. Labour pain is an expected and intricate part of childbirth [3]. The extent to which labour pain is experienced affects maternal psychology, labour progress and foetal well-being [4]. Physiological factors, such as uterine contractions and cervical dilatation, although essential, form part of labour and are the major contributors to the pain experienced during labour. Psychological factors such as stress, anxiety, fear, sense of loss of control and sense of abandonment also contribute to the level of severity of the pain experienced [1, 3]. There is also a wide range of factors which may influence labour pain, including personal, physical and medical characteristics associated with the woman in labour [2].

Labour pain intermittently begins with abdominal aches [5], pelvic pain [6] and backache [7]. Women who have undergone labour usually remember the high intensity of the pain [8] and largely classify labour pain intensity as mild, moderate and severe with increasing intensity from the onset of contractions to complete dilatation of the cervix [4, 9]. Women in labour, mostly express the intensity of the pains by crying and screaming [10, 11] as a coping strategy to such high intensity pain [1, 3]. It is reported in Nepal that, about 68.3% of women described labour pain as severe and over 86% of the women would want the pain to be relieved [12]. Many women desire to have a relief of pain during labour irrespective of colour, greed, social status, economic status or where they come from and this experience contributes immensely to their satisfaction with the experience of childbirth [13–16]. In Ghana some cultural groups label women as emotionally weak when they are not able to bear labour pain [4, 17] and as such, these women feel humiliated if other people knew they could not bear labour pains [4]. Many pregnant women have concerns about the pain they will encounter and the methods of pain relief that are available during labour [18, 19]. Labour pain relief is an important aspect of women’s health that has historically been neglected [20] mostly in the developing world. Most women are taught to endure pain rather than using medication as it is seen as a symbol of womanhood [17], and a sense of pride [4, 21]. This points out an inadequate antenatal education of women on the management of labour pain [13].

The need for pain relief in labour derives from the fact that the pains of labour are not known to be beneficial to the labour process [4, 13]. Non-pharmacological approaches such as emotional support to women from their partners [22], family members, professional or non- professional staff; help women to control labour pain [23]. Some women employ other non- pharmacological pain relief measures during labour such as breathing exercises, taking showers [24–26], assuming specific positions and ambulation to control pain [26, 27]. The need for effective labour pain management cannot be overlooked since labour pain makes the woman tired and this could affect her ability to effectively bear down (push with contractions) during the second stage of labour [5]. Even though the provision of emotional and psychological support is important in helping women go through painful childbirth, it is however unfortunate to note that in Ghana, support persons such as husbands and family members are not allowed into labour wards due to privacy issues [17], and institutional policies are equally said to prevent patient relatives from offering direct contact to their respective relatives labouring women [5, 17].

The important role played by midwives in offering caring support during the process of labour cannot be underestimated [3] especially in instances of positive interaction with women in labour [28], by showing caring and encouraging attitudes to women experiencing labour pain [29].

### Problem statement

Midwives just like other clinical staff sometimes underestimate the intensity of pain experienced by women in labour [9, 13], The cues used by midwives to differentiate pain intensities and qualities are similar to those used in other clinical settings, but may have limited discriminatory value as pain levels become severe [9]. Some pregnant women as well are not aware of the various labour pain management techniques and the means of alleviating it. Women's lack of appropriate knowledge about the risks and benefits of the various methods of pain relief can heighten anxiety [18, 19]. This study explored the experiences, expectation and perception of puerperal mothers on the use of non-pharmacological labour pain management techniques at the Adidome Government Hospital.

### Aim

The study explored the experience, expectation and perception of puerperal women on the use of non- pharmacological labour pain management at Adidome Government Hospital.

## Methods

### Design

An exploratory, descriptive, qualitative approach was employed which aimed at gaining an in-depth understanding of the perception, experiences of women during labour and the method used to alleviate pain. The phenomenological qualitative research design was specifically used to explore puerperal women experience of pain management used during labour in the Adidome government hospital of the volta region as the study focused on the commonality of pain experiences, and the women perceptions of these interventions. The choice of phenomenological design afforded the researchers the opportunity to explore a phenomenon in the real-life context of puerperal women experiences of care received during labour.

### Setting of study

The study was conducted in the Adidome District Hospital located in the capital of the central Tongu district of the Volta region, Ghana. The hospital serves as a referral centre to the peripheral health care facilities within the district and some parts of the greater Accra and Volta regions. The district population as at 2010 population and housing census was 59,411 with a growth rate of 3.5%. The growth rate is higher than both the regional and national growth rates of 2.5% [30]. Currently, the total bed capacity of the hospital is 108, of which the maternity ward has 22 beds. The hospital has a total of 240 staff comprising 160 clinical care staff. The hospital has ten units that included; maternity ward, female ward, male ward, children’s ward, emergency ward, eye clinic, mental health unit, dietetics department, pharmacy department and the reproductive and child health unit. The maternity unit like all the clinical sections in the hospital run three duty shifts that have a minimum of two staff (weekends and night duty) and a maximum of five (during week morning duty) during a shift. The maternity ward has an average daily admission of four as 80% of the admissions being labour cases. From the daily ward record book of 2018, the average number of deliveries in a day was four as 70% had a spontaneous vaginal delivery. The reproductive and child health unit provides antenatal, postnatal, family planning and child health services. The unit has an average daily attendance of 40, with 30% of the visits being postnatal visits.

### Study population, sample and sampling

The study population consisted of all puerperal women in the maternity ward and the post-natal clinic of the Adidome Government Hospital. A puerperal woman was described as a woman who had given birth within the last 42 days from the day of conducting the study. A purposive sampling technique was used in selecting participants and an in-depth individual interview session held. Data saturation was reached at the 17th interview when no new information was obtainable from participants even with probing [31]. The participants were purposely selected such that all related characteristics like age, religion, ethnicity, occupation, number of births and educational level were all included to allow for maximum variability sampling. Selected study participants were contacted directly and invited to the study venue and time to participate in the study after they sought care for post-natal care service.

### Pretesting and trustworthiness of the study

A pretest was conducted at the Volta regional hospital prior to the main study to test the tool’s trustworthiness and reliability, to allow for identification of any weaknesses in the plans whilst allowing time to rectify and necessary amendments equally made before the study was carried out.

To ensure trustworthiness of the study different approaches were adopted which included the use of the same interview guide throughout the entire study. An audit trail was kept for researchers to validate the methods undertaken in the study. A detailed description of the study setting, methodology (COREQ criteria were used) [32], and background of the sample were provided to ensure the transferability of the study findings to similar context. Furthermore, in-depth interviews ensured a full exploration of the women's experiences of non-pharmacological pain intervention used by midwives during labour. Concurrent data analysis (identified gaps to explore) ensured that non-pharmacological pain intervention experiences were further explored in subsequent interviews with successive participants. To ensure validity, data were returned to participants to cross check and verify their responses. Finally, the reporting of this study adhered to the 32 criteria recommended by the consolidated criteria for reporting qualitative research [32].

### Data collection tool

Data was collected through individual interviews. The interview was conducted using a self-developed interview guide that contained both open ended, some probes and closed ended questions. The closed ended questions allowed the researchers to ask specific questions and determine rating for pain and its management during labour. Apart from the demographic characteristics, some examples of closed questions that were used and sparse throughout the interview included; will you say the pain you experience was moderate, severe or very severe; how did you feel about the attitude of the midwives, was poor, very poor, satisfactory or very satisfactory; tell me exactly how you managed the pain during labour etc. The open-ended questions on the other hand allowed the participants to express and describe in detail their experiences of the labour process. Some examples of open-ended questions used included how will you say the pain experience labour was; how did you express labour pain; how different was your experience of pain in the last labour to your previous knowledge regarding labour pain; what were some of the techniques you adopted to relief labour pain etc. Also, during the interview process, the researcher was allowed to ask for questions (probe) for clarity of the expressed statement or made by the respondent to describe in detail. Generally, the probes that were used in this study were not predetermined and originated from the researchers experiences and the need to seek clarity.

### Data collection and analysis

The three people (one with a masters of philosophy degree and the other two with bachelor degrees) that conducted the interviews were workers from the University of Health and Allied Sciences, Ho, Ghana. Prior to the conduct of the study, the three researchers (two males and a female) received two days training on the study tool, interviewing skills, consenting procedures and ethical issues associated with research. The puerperal women (after they gave consent- all women contacted consented to participate in the study) were taken to a secluded room for the individual interview session to maintain confidentiality and privacy. Questions on the interview guide were based on the objectives of the study as follow up questions were asked based on the women’s response. The data collection started in March, 2018 and ended in June, 2018 and conducted during the day. Each interview session lasted 25 to 30 min. Three trained researchers conducted each interview session. Field notes were taken during each interview session. After the interview session on each day, the responses that were audio recorded were transcribed verbatim and the transcript stored in Microsoft word format together with field notes. Participants were given labels (A, B, C, etc.) so as not to link directly their responses to the individual study participants.

In data analysis, thematic analysis was engaged that embraces three interrelated stages, namely data reduction, data display, and data conclusion [33]. Also, the process of thematic analysis involved construing through textual data, identifying data themes, coding the themes, and then interpreting the structure and content of the themes [34]. In using this scheme, a codebook was first established, discussed, and accepted by the authors. Theme codes were then created within NVivo software (version 11) using the codebook. Line-by-line coding of the various transcripts was performed as either free or tree nodes. Double coding of each transcript was carried out by two of the authors. Coding comparison query was used to compare the coding and a kappa coefficient (the measurement of inter-coder reliability) was generated to compare the coding that was conducted by the two authors. The themes that were identified and presented included puerperal women’s perception of severity of labour pains, means puerperal women expressed labour pains, perception of midwives’ attitude towards pain management during labour, labour pain-relieving techniques adopted by women in labour, the provision of information regarding labour pains, parturient women’s involvement in pain management, approval of parturient women regarding labour pain management and experiences of labour among women base on the number of previous births The views expressed by participants were used as quotes to buttress the points made and also cardinal views were summarised and presented as simple frequencies or proportions.

### Ethical considerations

Ethical clearance was obtained from the scientific and ethics committee of the Institute of Health Research of the University of Health and Allied Sciences, Ho- Ghana. Permission was also sought from the management of Adidome Government Hospital and the central Tongu district health directorate. Before participants were interviewed, each of them was taken through the informed consent process and the objectives of the research explained to them in line with the requirement of the ethics review board and the Helsinki declaration for conducting research. Involvement of participants in the study was fully based on their voluntary participation in the study as participants gave both written and verbal consent to participate. Study participants were also having the option to withdraw from the study anytime they so wished to do so.

## Results

### Socio-demographic characteristics of participants

The puerperal women (11.8%) were under the ages of 20 years as the majority of the women were unemployed (52.9%) as depicted in Table 1. On the level of education, the majority (52.9%) had attained secondary school level while 58.8% were married. The puerperal women (88.2%) were Christians and Ewe (94.1%) by ethnicity. The women (64.71%) had two pregnancies and five pregnancies (5.9%). All the women had their deliveries in the hospital.

**Table 1:** Socio-demographic and birth history of puerperal women

Variables	Responses	Number of responses	Percentages
Age	Less than 20	2	11.8
	20–29	12	70.6
	30–39	3	17.6
Employment status	Unemployed	9	52.9
	Civil/government work	1	5.9
	Self/privately employed	7	41.2
Level of education	Uneducated	1	5.9
	Basic	6	35.3
	Secondary	9	52.9
	Tertiary	1	5.9
Marital status	Single	10	58.8
	Married	7	41.9
Number of pregnancies	One	3	17.7
	Two	11	64.7
	Three	2	11.8
	Five	1	5.9

### Puerperal women’s perception of severity of labour pains

The researchers ask participants to describe the nature of labour pains they experience in the last delivery. The women expressed their labour pain as very severe pain while others expressed their labour pain as severe pain moderate pain. The very severe labour pain was rated to be unbearable, severe was too painful, but bearable while moderate pain was compared to the pain the participant had previously experienced.

“It is one of the most severe forms of pain one can ever experience. This pain was actually unbearable. I think it is the worst type of pain ever to be experienced by anybody. There is no amount of pain that can be used to compare to the severity of this type of pain during labour. It is so piercing!” (Participant C, 22 years).

*“The pain was so *severe and unbearable; I did not know what to do but to only bear…” (Participant E, 36 years).

“…good thing about the pain is that it comes and goes, so you will find some relief, but when the pain* is on, there is nothing to be compared to. It is so severe and generally can only be said to be unbearable and the severest form of pain.”* (Participant N, 29 years).

The study also revealed that women indicated their labour pain lasted less than 12 h. The actual period that the pain commenced till labour was complete varied from each woman.

*“The pain *became severe in the morning and by the evening I had given birth. But in the peak of the pain, nothing could be comparable” (Participant M, 27 years).

“For the pain it lasts the entire duration of the labour, because the pain goes and comes. But I can say that the pain lasted the whole day” (Participant A, 27 years).

“For me, contrary to what other women say and experience, I had the labour start like 1 pm and* by 10 pm I had put to birth. But for the pain it was so so so severe”* (Participant F, 19 years).

### Means puerperal women expressed labour pains

Labouring women used various strategies in managing severe labour pains. This strategy ranges from making noise to adopting comfortable body positioning and snapping of figures. The women expressed their labour pain by shouting and walking around, crying and screaming and staying calm and snapping their fingers. This way of expressing the pain was described by the women as self-comforting.

*“I can’t remember, *but I am told I was shouting and calling my husband, I think doing this actually helped me to endure the pain. Honestly, it was really a painful experience” (Participant D, 25 years).

“I was so restless and had to walk from one place to another. I just walked around the labour wards, especially when the pain became severe. I think the walking around the facility* actually helped me to adjust better. It made me feel the labour less compared to my previous labour.* (Participant H, 29 years).

*“There is nothing to be done to alleviate the pain. I walked and sometimes I scouted by the bed. I was just restless. I went to the washroom many times hoping for relief. In fact, I think I did everything as long as the pain was that much severe”* (Participant G, 24 years).

### Perception of midwives’ attitude towards pain management during labour

The puerperal women had varied descriptions for the attitude of the midwives during labour and their general predilection towards effective pain management. On the aspect of the women’s perception of the midwife's attitude, most of the women indicated they were satisfied or very satisfied with the midwives' attitude towards pain management. Others indicated the attitude of the midwives was poor or very poor. Women gave approval for the positive attitude of midwives during the labouring process. The attitude of the midwife related to her empathy and desire to institute measures that can alleviate labour pain. Midwives that remained focused on this course were described as having a satisfactory attitude and those who did not express a desire to help were described as having a poor attitude or low satisfaction.

*“I *must say, contrary to my prejudice about midwives’ attitude in the labour ward, the one who attended to me was so caring, sometimes when I remember some of the questions, I asked her, yet she had the patience to respond, I must say that she was a God sent” (Participant B, 18 years).

“The midwife that attended to me in this last pregnancy was an angel. She explained to me so well what to expect, she supported me when I needed it most and also allo*wed my husband to stay with me in the larger part of the labour period, I honestly feel very cared for”* (Participant K, 28 years).

However, some of the women had a contrary view of the attitude of the midwives that attended to them during their last labour process. As they indicate they were not satisfied with the attending midwives’ attitude, participants tried to ascribe reasons for such behaviours.

*“The least *talk* of the midwife the better. I think that day she was so overwhelmed with the number of women in labour. We were four women under her care and she was ‘kind of confused’. Even If you called her, she spent so much time before she came to one’s attention. I don’t hope to have such an experience again. For me this last labouring experience was actually a nightmare”* (Participant Q, 23 years).

*“I almost gave birth in the washroom, when I said the baby was coming, they wouldn't listen, they were just there watching television and talking. A midwife even asked me if I was the first person to go into labour”* (Participant L, 26 years).

### Labour pain-relieving techniques adopted by women in labour

The participants use various techniques to reduce pain during the last labour. The mothers revealed that they employed the technique of breathing through their mouths, others engaged in a conversation, engaged in diversionary therapy & reassurance. These techniques that are adopted by women in labour were taught them during the antenatal clinic (ANC) visits.

“*The *midwives in the ANC had taught me that since this was my first experience, when the labour set in I should inhale through my nose and breathe out through pursed lips slowly. True, it was reiterated to me during labour and honestly, it was actually very helpful” (Participant E, 36 years).

“I was told during* ANC that when labour sets in, I should have someone I care for and love to stay with me. When I was in labour because it was a private facility, my husband was allowed into the ward because we were the only people. My husband was so supportive, sometimes I feel sorry for his experiences that day”* (Participant O, 25 years).

This notwithstanding, some women said nothing was done to manage their pain. The study also looked at other methods that were used to relieve the labour pains of the women. The majority of the women said they received supportive care from their relatives and the midwives. Some women described the labour process as a natural process that is expected to be painful and in light of this, they could not phantom any non-pharmacologic method that could be instituted to alleviate the pain of labour. Such women had little or no expectation of pain management during labour.

*“I cannot *remember* what was done specifically because I was in pain. I do not know however what they could have done about it anyway. I just endured the pain because I understood it as a normal process that will help bring forth a life”* (Participant B, 18 years).

*“For me labour is a natural process that ought to be painful. I do not think I expected anyone to help me bear the pain, I had already made up my mind that it’s a painful process and true to my thought it was actually that painful. I could only bear it. The pain is enshrined by God* (Participant F, 19 years).

The change in body position and the nursing interventions instituted by midwives also aided in improving pain relieve among parturient women. Some indicated they received other pain-relieving methods such as intravenous fluid, bathing and laying on sideways. Key to the interventions were the fact that some support was instituted too late when the services were not needed or no more anticipated. Also, others identified some pharmacologic interventions that were instituted to support the management of the pain.

“*For me the labour lasted *two long and was so painful. So, when the doctor came on rounds, I complained and was given some medications and intravenous infusions” (Participant M, 27 years).

“I was given an injection only at* the point I could not bear the pain any longer. For me that injection was of no use because shortly after that I was put to birth”* (Participant J, 29 years).

*“Was not given any *specific* medicine”* (Participant G, 24 years).

### The provision of information regarding labour pains

Indicating if the midwives provided enough information regarding labour pains, the women had varied opinions. Some of the women indicate that the information provided by midwives regarding pain management during the labour was insufficient. Others indicated that they were not given information at the point of labour, but were given education during the ANC about the labour process and cervical dilations, contractions and bearing down. During labour information given specifically of the expectations throughout the process and how long it will last influence positively the woman’s ability to bear labour pains.

“*I think that the midwives need to always tell the labouring women what to expect, compared to my first labour experience, in the last one the midwife told me I will give birth in about seven hours’ time, right at the time of arrival, so I could predict the progress of the pain.”* (Participant C, 22 years).

*“I was told to take deep breaths when the pain comes, I think that was so supportive in alleviating *the* pain to some extent”* (Participant O, 25 years).

Participant Q, 23 years.

### Parturient women’s involvement in pain management

The study revealed that the women were not involved in decision making about labour pain management. The nature of the involvement included education, information giving and expressing of empathy, allowing the presence of family members like the husband in the labour room and listening to the concerns expressed by the women by the midwife.

*“I received some education on what to do when labour pain becomes severe during ANC services. Actually, the *midwife* was also very empathetic and seems to understand the pain I was experiencing”* (Participant I, 38 years).

*“The midwife told my husband to massage my back when the pain is severe and he did, so it made me feel *better*. I think the presence of my husband in the labour room was supportive. However, I do not think that if it was a government owned facility, I would have benefited from this”* (Participant O, 25 years).

Some of the women, however, had different experiences when it came to their involvement in pain management during labour. They contend the care that was given was appalling and did meet their basic expectations as some could not identify anything that was done specifically to alleviate pain during labour.

*“No one asked me about how I felt. From the behaviour of the midwife, I wondered at a point if she is aware *that it was painful. Her behaviour was very appalling” (Participant A, 27 years).

“I cannot immediately identify anything* they did for me because I was in pain. I think I only did things to alleviate the pain by myself”* (Participant J, 29 years).

### Approval of parturient women regarding labour pain management

On the aspect of approval with labour pain management by midwives, the women approved of the pain management they received from midwives while some did not approve of the labour pain management they received from midwives.

*“My experience in the labour ward was one I do not wish any woman in labour to go through. How could I have been satisfied when I was treated like a machine that is just supposed to bear the pain because labour is a natural phenomenon?”* (Participant Q, 23 years).

*“For me labour was my worst nightmare and the midwife even made it worse for me. It’s like the midwives are trained to care about nothing. Irrespective of the amount of pain one is bearing they just look on unconcerned”* (Participant M, 27 years).

*“I can say compared to my previous experiences; this one was fairly welcoming. The midwives in the labour ward have now changed. They were caring and supportive”* (Participant D, 25 years).

*“Having to give birth in a private hospital was a good experience for me. I had the luxury to do anything and more so I was the only woman in labour at the time. I received the maximum support from the midwives and all the care professionals”* (Participant O, 25 years).

The women indicated the midwives did their best in managing their labour pains as others showed the midwives could do better. Finally, the women alleged the care given them by the midwives was good as others showed it was bad and the remainder showed the midwives could do better.

### Experiences of labour among women base on the number of previous births

For those who were first labouring women expressed pain as very severe while some reported moderate pain.

*“Contrary to what I have always heard and thought, I think the pain was manageable, indeed I was *still expecting the worst when the midwife asked me to move to the delivery room because I was due” (Participant B, 18 years).

“I have very severe pains only about three hours of the birth. Yes, labour is painful, but it’s not that the pain persists throughout the period one is labouring. The intensity, frequency and duration* of the pain only increases as the labour itself progresses. I think the worst case is in the last hours preceding the birth itself”* (Participant K, 28 years).

For those who had attended labour two times, the majority reported severe pain and others indicated moderate pains. Also, those attending labour for the first time were satisfied with the midwives’ attitude during labour pain care. Finally, those who attended labour once or twice gave a good general comment about the care given them.

## Discussion

This study assessed the labour pain management that was adopted by women in labour in Adidome Government Hospital of the Volta region of Ghana. The ability of women to institute pragmatic pain management influenced directly the total outcome of a labour experience. It was found that the women expressed very severe pains during labour and having pains lasting for twelve or more hours. In Nepal, about 68.3% of women described labour pain as severe and above 86% of the women would want the pain to be relieved [12]. Culture, economic and social antecedents have an influence on women's ability to experience, grade and express pain. Women adopt various strategies and methods to acclimatise to pain as mothers’ express labour pains by shouting and walking. This type of main management strategies that have been adopted by women was not new as in several other studies, women showed that they adapted to labour pain by walking, screaming and or shouting [10, 11]. This type of pain management method may not be strange, but noise pollution can be an associated distress to workers in the labour ward and it is imperative that in the African society, labour wards are fitted with sound proof gadgets as women will feel more secure and safe to scream or produce any sound they so wish to. The sound proof may include installation of sound curtains or thick blankets, use of bookcases to make walls thicker, mounting shaky items, installation of door sweep and use of acoustic wedges panels.

Other forms of pain management strategies adopted during labour included women engaged in deep breathing exercises, chatting with other people and relatives, diversion therapy, reassurance, taking a shower, assuming side lying positions, and receiving intravenous therapy. The women contend the institutionalization of these methods aided in the effective management of pain especially during labour. The use of these methods of pain adaptation has been reported by other researches [24–27]. Emotional support to women from their partners, family members and professional staff helped women to adapt to pains better. It is imperative that midwives are able to build a lasting cordial rapport between them and the woman in labour so as to serve a strong reassuring support and alleviate the pain women experience. The role played by good interpersonal relationships and the presence of a trusted person has been shown to improve women's pain adoption ability [13, 22, 23].

The women approved of the care that was given by the midwives towards labour pain management. The level of women’s perception of midwives’ attitude influences essentially their total birth outcomes. These perceptions influence the nature and extent of the pain they experience during the birthing process. It is imperative that midwives institute pragmatic protocols during labour to ensure that women feel comfortable and relaxed. Midwives need to build cordial mutually beneficial relationships between the practitioner and the woman in labour and provide adequate information on the measures they have instituted to alleviate pain. Some women who have undergone labour in the Adidome government hospital reported that no labour pain alleviating strategy was rendered to them. It is important that as women journey through childbirth, they are provided with adequate related information about labour and the processes that are necessary in alleviating pain, especially during ANC. This confidence was enabled through a relationship of trust that developed between women and midwives, and the value of listening to women’s experiences. These experiences enhanced women’s ability to overcome fears and self-doubt about coping with pain and led to feelings of pride, elation, and empowerment after birth [28].

In some instances, labouring women requested pharmacologic pain relief that included the use of intravenous therapy. In Nigeria only 34.1% of respondents were aware of their right to labour analgesia. The vast majority of pregnant women in the present setting do not know that they have the right to request and be given pain relief during labour. Most women indicated their support for labour analgesia and the desire to demand it, indicating that women in that study setting have been “suffering in silence” during labour [13]. Most pregnant women are not aware of the various labour pain management techniques and the means of alleviating pain and because of this lack of appropriate knowledge about the risks and benefits of the various methods of pain relief, can heighten anxiety among these women [13, 18, 19]. It is imperative that pregnant women are involved in the care process, especially when they are in labour. When women are not involved in pain management, it has the potential to encourage paternalism in labour care—a situation in which the opinions and wishes of the caregivers hold sway, with little or no consideration for the wishes of the parturient. The principle of autonomy, which demands the respect of maternal wishes and choices, is breached by caregivers’ denial of maternal requests for labour analgesia [13]. The same goes to the principle of justice, which demands fair treatment for all patients [13]. 

The empowerment of women can be attained when adequate measures are instituted during ANC, to ensure that pregnant women received the required information regarding pregnancy and childbirth. In London, support from caregivers who have a strong belief in women’s ability to give birth without pharmacological pain relief has been identified as an important feature of midwifery practice [28]. In using the principle that the same midwife in the ANC attends to women in labour, in London, it's reported that women reflected positively on how, throughout pregnancy and labour, their midwives promoted a sense of their ability to cope with the challenge of labour pain [28]. Inherent in the Ghanaian system will be the challenge of inadequate midwives. However, the need to give standardized information to women during ANC is imperative as this will ensure that all women have enough and adequate knowledge on pain management.

This study highlights the views of puerperal women’s experiences of labour and how pain was managed. Study participants had to recall a past experience of an unpleasant situation and response could have been blurred by the desire not to relive such experiences or generally low sense of judgement influenced by the pain during labour. Also, a qualitative sampling method of using only 17 participants through purposive sampling in a single health facility may pose a risk for over generalisation of study findings in the larger population. Irrespective of this limitation, this study particularly highlights the important role of pain management among parturient women as further studies are required to measure and validate some of the pain adaptation strategies that were adopted.

## Conclusion

Women reported severe pains during labour lasting for more than 12 h. Women also approved of the care rendered to them as they perceived the midwives to be kind and attended to their needs. The presence of the woman’s significant other (e.g., husband) in the labour room during labour was seen to improve the women’s pain bearing ability. It was also identified that women used various strategies in pain management, including shouting, screaming and wailing. As this can be seen as a common pain adoption measure, labour units can be fixed with noise proof gadgets that allow women to express themselves without the risk of fear of believing that people outside can hear. This sound proof can be done through installation of sound curtains or thick blankets, use of bookcases to make walls thicker, mounting shaky items, installation of door sweep and use of acoustic wedges panels. It is important that midwives institute pragmatic protocols in the labour ward that ensure a relaxing atmosphere for women in labour, respond to the sensitivity and specificity of labouring women needs and when possible significant others of the labouring women allowed into the labour room. During ANC, expectant mothers should be educated more on some non-pharmacological methods of pain management so as to reduce anxieties and improve decision making ability.

## Data Availability

Not applicable.

## Abbreviations

COREQ: Consolidated Criteria for Reporting Qualitative Research
ANC: AnteNatal Clinic
